# *Strongyloides stercoralis* seroprevalence in Vietnam

**DOI:** 10.1017/S0950268817002333

**Published:** 2017-10-17

**Authors:** NGUYEN THI NGOC DIEP, PHAM QUANG THAI, NGHIEM NGUYEN MINH TRANG, JULIA JÄGER, ANNETTE FOX, PETER HORBY, HOANG VU MAI PHUONG, DANG DUC ANH, LE THI QUYNH MAI, H. ROGIER VAN DOORN, BEHZAD NADJM

**Affiliations:** 1 Oxford University Clinical Research Unit and Wellcome Trust Major Overseas Programme, Hanoi, Vietnam; 2 National Institute of Hygiene and Epidemiology, Hanoi, Vietnam; 3 Department of Microbiology, Immunobiology & Genetics, University of Vienna, Austria; 4 Peter Doherty Institute for Infection and Immunity, Department of Microbiology and Immunology, University of Melbourne, Victoria, Australia; 5 Center for Tropical Medicine and Global Health, Nuffield Department of Clinical Medicine, University of Oxford, Oxford, UK

**Keywords:** Helminths, serology, strongyloidiasis, Vietnam

## Abstract

Strongyloidiasis is a neglected tropical disease caused by the roundworm *Strongyloides stercoralis* affecting 30–100 million people worldwide. Many Southeast-Asian countries report a high prevalence of *S. stercoralis* infection, but there are little data from Vietnam. Here, we evaluated the seroprevalence of *S. stercoralis* related to geography, sex and age in Vietnam through serological testing of anonymized sera. Sera (*n* = 1710, 1340 adults and 270 children) from an anonymized age-stratified serum bank from four regions in Vietnam between 2012 and 2013 were tested using a commercial *Strongyloides ratti* immunoglobulin G ELISA. Seroreactivity was found in 29·1% (390/1340) of adults and 5·5% (15/270) of children. Male adults were more frequently seroreactive than females (33·3% *vs.* 24·9%, *P* = 0·001). The rural central highlands had the highest seroprevalence (42·4% of adults). Seroreactivity in the other regions was 29·9% (Hue) and 26·0% and 18·2% in the large urban centres of Hanoi and Ho Chi Minh City, respectively. We conclude that seroprevalence of *S. stercoralis* was high in the Vietnamese adult population, especially in rural areas.

*Strongyloides stercoralis* is a soil-transmitted intestinal nematode that causes strongyloidiasis. It has a worldwide distribution, with likely more than 30–100 million people infected [1]. The wide range in the estimates of the burden of disease is likely related to the lack of studies from areas where the infection is common and debate as to what the health impacts of chronic infection are. Infection is more frequent in areas with poor hygiene and warm, humid conditions. The majority of infected people are asymptomatic with chronic infection. Unlike other human intestinal nematodes, *S. stercoralis* has the capacity to complete its entire lifecycle in the human host through autoinfection, thereby maintaining infection for many decades. In patients who become immunosuppressed, for example, through corticosteroid therapy, autoinfection can go unchecked and large numbers of larvae may disseminate widely and cause a hyperinfection syndrome, which can be fatal [1].

Vietnam is a developing tropical country where an agrarian-based economy, which featured widespread use of night soil (human manure as fertilizer), is transitioning into an increasingly urban, manufacturing economy with rapid improvements in healthcare and lifespan. This transition comes with an increase in diseases such as chronic obstructive pulmonary disease, cancers and chronic diseases and their associated immunosuppressive therapies. Rural to urban migration over the last 15–20 years has been dramatic. Mass deworming campaigns typically target children and women of childbearing age in rural areas and employ strategies of limited efficacy against *Strongyloides* worms, such as single-dose benzimidazoles [2]. As a consequence of these changes, *Strongyloides* hyperinfection syndrome is likely to become increasingly relevant.

Very little data are available on the prevalence and distribution of *S. stercoralis* in Vietnam. In a recent meta-analysis reviewing the prevalence of *S. stercoralis* in Southeast Asia over the last 25 years, the prevalence of *S. stercoralis*, based on multiple published studies, ranged from 36% in Malaysia to 24·5% in Cambodia [3]. Only a single study from Vietnam was eligible for inclusion, giving a reported prevalence of 0%. This study likely grossly underestimated the prevalence as it used single untreated stool microscopy and agar plate culture in a cohort of children in the Mekong delta region of Vietnam [4].

Diagnosis of strongyloidiasis has traditionally been based on microscopy (Baermann test) to detect *S. stercoralis* larvae in faeces, supplemented by culture in some research studies. However, patients with chronic *Strongyloides* infection usually have a low parasite load and irregular shedding making microscopic diagnosis insensitive. Serological methods have higher sensitivity in a chronically infected population [5]. Enzyme-linked immunosorbent assay (ELISA) to detect human immunoglobulin G (IgG) against a crude extract of infective larvae is one of the most widely used techniques for the diagnosis of strongyloidiasis, with sensitivity rates between 73% and 100% [6].

Our objective was to provide baseline data for Vietnam and inform healthcare authorities on the proportion of patients at risk for developing preventable complications of immunosuppressive therapy. To this end, an adult population representing those most likely to receive immunosuppression was selected. In order to understand whether ongoing transmission was occurring in those that would not have been exposed 2–3 decades ago, prior to changes in farming and sanitation, a second, paediatric population was selected. We therefore used a commercial ELISA to test anonymized serum samples to estimate the prevalence of *Strongyloides* infection in the Vietnamese population and compare this between sexes, four regions in Vietnam, and between adults over 40 years and children aged between 13 and 15 years.

Sera were selected from a serum bank established according to the published guidelines from the European Siro-Epidemiology Network for the surveillance of influenza immunity [7]. Briefly, sera were collected from residual samples taken for biochemical testing from patients attending four hospitals in four regions across Vietnam: Thanh Nhan Hospital in Hanoi (north), Hue Central Hospital in Hue (central coastal), Dak Lak General Hospital in (central highlands) and Gia Dinh Hospital in Ho Chi Minh City (HCMC, south). Residual blood samples from all wards except specialized infectious diseases, HIV and oncology wards that were sent to the diagnostic laboratory for routine testing were collected in an age- and sex-stratified manner between December 2012 and November 2013. Samples were deduplicated, anonymized and stored centrally at −20 °C until use.

Assuming a seroprevalence of 25% among older adults in a high prevalence region and 15% prevalence in a low prevalence region, a sample of 335 sera per region would provide 90% power to detect this difference at an *α* of 0·05 (1340 adult sera total). A sample of 270 children's sera would permit demonstration of a difference between adult samples at an average 20% prevalence and child samples at an estimated 10% prevalence with similar power and *α*.

Sera were selected randomly from the serum bank, stratified by age group, sex and location. Based on the sample size calculation above, sera from a total of 1340 adults (40–70 years old) and 270 children (13–15 years old) were tested in this study (335 adults and 67 or 68 children per hospital). A commercially ELISA kit (*Strongyloides ratti*, Bordier Affinity Products SA, Crissier, Switzerland) was used to determine IgG antibodies against *Strongyloides* following the manufacturer's instructions. The Bordier ELISA uses the antigenic similarity between *S. ratti* (a related helminth that infects rats) and *S. stercoralis*.

The median and mean ages of the patients whose sera were tested were similar in all four sites (Supplementary Table). Of 1340 adult samples, 390 (29·1%) were positive. Sera from Dak Lak showed the highest proportion of positives (142/335, 42·4%), followed by Hue (100/335, 29·9%), Hanoi (87/335, 26%) and Ho Chi Minh City (61/335, 18·2%). The seroprevalence was higher among males than females (223/670, 33·3% *vs.* 167/670, 24·9%, *χ*^2^ *P* = 0·001). There was no significant difference in the proportion of positive sera between the three age groups in adult patients (Table 1).

**Table 1.** Seroreactivity by age group and location

Age group	Dak Lak n/N (%)	Hue n/N (%)	Hanoi n/N (%)	Ho Chi Minh City n/N (%)	Total n/N (%)
Child total (13–15 years)	3/67 (4·5)	5/67 (7·5)	1/68 (1·5)	6/68 (8·8)	15/270 (5·6)^†^
40–49 years	43/102 (42·2)	23/108 (21·3)	27/110 (24·5)	23/118 (19·5)	116/438 (26·5)
50–59 years	52/115 (45·2)	35/111 (31·5)	36/113 (31·9)	19/112 (17·0)	142/451 (31·5)
60–69 years	47/118 (39·8)	42/116 (36·2)	24/112 (21·4)	19/105 (18·1)	132/451 (29·3)
Adult total (40–69 years)	142/335 (42·4)	100/335 (29·9)^†^	87/335 (26·0)^†^^,^^‡^	61/335 (18·2)^‡^	390/1340 (29·1)^†^

* *χ*^2^ Test *P* < 0·001 for comparison of child total and adult total.

† *χ*^2^ Test *P* = 0·263 for comparison of Hanoi adult total and Hue adult total.

‡ *χ*^2^ Test *P* = 0·015 for comparison of Hanoi adult total and Ho Chi Minh City adult total.

The proportion of positive sera among children was much lower than in adults, ranging from 6/68 (8·8%) in Ho Chi Minh City to 1/68 (1·5%) in Hanoi and no significant differences between sites were found. Of the 270 children samples across all sites, 15 (5·5%) were positive.

In this cross-sectional assessment of strongyloides seroprevalence across Vietnam, we showed a high proportion of seroreactivity among adults. Proportions varied substantially by geographical region from 18% in Ho Chi Minh City to 42% in Dak Lak. Our results showed a larger proportion of adults than children to be positive, either reflecting progressive cumulative exposure with age or improving hygiene conditions. Further, given that the hospitals were based in cities, a large proportion of adults may have had exposure to *Strongyloides* in rural areas prior to urban migration, whilst children raised in the cities had minimal exposure. The study was not powered to explore differences in seropositivity between sites among children; a larger sample size would be needed to establish if there were significant differences. A larger proportion of male adults than female adults was found, which may reflect differences in professional daily activities and hygiene practices or the targeting of females with health interventions. Both findings are in keeping with other serological studies and other studies using parasitological methods [8]. The rates of seropositivity are also in keeping with the estimated prevalence in neighbouring countries such as Cambodia (21–30%) and Laos (>30%) [3].

One other recent study, completed after the publication of the meta-analysis referred to earlier, reported strongyloides seropositivity of 7·4% in Vietnam, summarising diagnostic results from samples sent for parasitology diagnostics to a commercial laboratory in HCMC, Vietnam [9]. The DRG Instruments Strongyloides ELISA (DRG Instruments, Springfield, Illinois, USA) was used here. The lower rates of seropositivity, even though samples were sent in with a specific request for parasitological testing, are likely the result of the different demographic (urban population, private clinic) included in this study.

The selection of sera from hospitals may have created a bias towards a higher estimated prevalence, as certain conditions that are more common among hospital patients, such as alcoholism, have been linked to *Strongyloides* infection [8]. In contrast, a negative bias may have been created by the fact that all hospitals were located in urban settings with potential under-representation of individuals from rural, more remote settings.

The use of serology opens these data to criticism that the results may represent cross-reactivity or past infection. With regard to results representing past infection, studies of patients treated for *Strongyloides* based solely on a serological diagnosis, demonstrate that almost all patients have a significant fall in titres over time, usually to negativity [10]. This strongly suggests that these patients have infection with *Strongyloides* or another cross-reacting helminth, rather than past infection. Regarding cross-reactivity, the test used has been shown to have specificity of over 95%, with significant cross-reactivity reported only to filaria [6], which has very limited occurrence in Vietnam [11]. Other studies have shown lower specificity [12], and the manufacturers own datasheet suggests a specificity of 77% in patients with other helminth infections [13]. Even using these lower estimates of specificity, whilst assuming 100% sensitivity, chronic *Strongyloides* infection would be common in the adult population with between 14% and 32% of adults likely to be harbouring the infection. What is clear is that linkage of these serological data with case matched, high-quality parasitological data from large-scale studies in endemic areas is necessary to establish the burden of strongyloidiasis. The methodology used in this study could then be used to generate more accurate national and global figures.

In conclusion, infection with *S. stercoralis* is common among Vietnamese adults, with higher seroprevalence in rural than urban settings. The study revealed that gender and age are risk factors for *Strongyloides* infection in Vietnam. Thus, a significant proportion of patients receiving immunosuppressive therapy may be at risk of developing hyperinfection syndrome. In the USA, the Centers for Disease Control and Prevention (CDC) considers that refugees from high-risk populations should receive presumptive treatment for strongyloidiasis [14], whilst screening and treatment prior to induction of immunosuppression is recommended for those with history of travel to endemic areas [15]. Further work to establish the impact of *Strongyloides* on health and the correct management strategies for endemic countries in economic transition is warranted.

## Supplementary material

For supplementary material accompanying this paper visit https://doi.org/10.1017/S0950268817002333. click here to view supplementary material
