# Supplementary material for: Strongyloides stercoralis seroprevalence in Vietnam
Source: Epidemiol Infect. 2017 Oct 17;145(15):3214–8. doi: 10.1017/S0950268817002333 (PMC7116469; doi:10.1017/S0950268817002333)
Supplement: Supplementary file 1 [file S0950268817002333sup001.docx]

Supplementary Table. Strongyloides in Vietnam.

|  | **Dak Lak**  **(n=335)** | **Hue**  **(n=335)** | **Ha Noi**  **(n=335)** | **HCMC**  **(n=335)** |
| --- | --- | --- | --- | --- |
| Mean (SD) | 55.6 (8.4) | 55.3(8.6) | 55.3(8.2) | 54.6(8.5) |
| Median (IQR) | 57.5  (42.5 – 67.5) | 57.5  (42.5 –67.5) | 57.5  (42.5 -67.5) | 52.5  (42.5 – 67.5) |

**Supplementary Table.** Age summary of tested adult sera by location
